# Longitudinal association of C-reactive protein and Haemoglobin A_1c_ over 13 years: the European Prospective Investigation into Cancer - Norfolk study

**DOI:** 10.1186/s12933-015-0224-1

**Published:** 2015-05-22

**Authors:** Sara Ahmadi-Abhari, Stephen Kaptoge, Robert N Luben, Nicholas J Wareham, Kay-Tee Khaw

**Affiliations:** Department of Public Health and Primary Care, University of Cambridge, Cambridge, UK; Medical Research Council (MRC) Epidemiology Unit, Institute of Metabolic Sciences, Cambridge, UK; Department of Public Health and Primary Care, University of Cambridge, Strangeways Research Laboratory, Worts Causeway, Cambridge, CB1 8RN UK

**Keywords:** Inflammation, Type 2 diabetes, C-reactive protein, Haemoglobin A_1c_, Longitudinal Study

## Abstract

**Background:**

Type-2 diabetes is associated with systemic inflammation and higher C-reactive protein (CRP) levels. However, the longitudinal association of CRP and haemoglobin-A_1c_ (HbA_1c_) has not been described in large prospective studies. Understanding such associations may shed light on the role of inflammation in development of type-2 diabetes and its complications such as cardiovascular diseases.

**Methods:**

EPIC-Norfolk is a cohort study of men and women aged 40–79 years at time of recruitment (1993–1997). Serum CRP (mg/l) was measured using a high-sensitivity assay at baseline and 13-years follow-up. HbA_1c_ (%) was measured at baseline, 4, and 13 years. Participants were excluded if they were diagnosed with diabetes or were taking diabetes medication. Data on at least one measurement of CRP and HbA_1c_ was available for 14228 participants (55 % of the cohort).

**Results:**

In the cross-sectional analysis of baseline data, a 1-SD higher log_e_-CRP (about three-fold higher CRP) was associated with 0.06 (95 % CI 0.04, 0.08) higher HbA_1c_ (%) adjusted for potential confounders. In longitudinal analysis using multivariable linear mixed models, change in CRP over 13 years was to a similar extent positively associated with increase in HbA_1c_, such that 1-SD higher longitudinal change in log_e_-CRP was associated with 0.04 (95 % CI 0.02, 0.05) increase in HbA_1c_.

**Conclusion:**

In this study we found longitudinal observational evidence suggesting that increase in systemic inflammation is associated with an increase in HbA_1c_ and thus systemic inflammation may have a role in development of type-2 diabetes and its complications.

## Background

Serum C-reactive protein (CRP) is a marker of systemic inflammation and has been shown to be associated with incident type 2 diabetes [1,2]. Patients with type 2 diabetes are also at risk of developing micro-vascular and macro-vascular complications such as diabetic retinopathy, nephropathy, neuropathy, atherosclerosis, and cardiovascular diseases. The potential etiologic role of chronic systemic inflammation in the pathogenesis of type 2 diabetes and in the link between diabetes and micro-and macro-vascular complications is not well known.

Haemoglobin A1c (HbA_1c_) concentrations in patients with diabetes are associated with risk of developing micro- and macro-vascular complications, and is thus used as the “gold standard” for monitoring glucose control in patients with type 2 diabetes [3]. HbA_1c_ has also been advocated to be used as a marker for diagnosis of type 2 diabetes [3]. Therefore, understanding the association between markers of systemic inflammation (such as CRP) and haemoglobin A_1c_ may help better understand the potential role of inflammation in the development of type 2 diabetes and in the mechanisms involved in development of complications in patients with diabetes. A positive cross-sectional association between CRP and HbA_1c_ has been reported previously [4-7]. However, the temporal association of CRP and HbA_1c_ over time has not been described. Exploring the longitudinal association between changes in CRP and changes in HbA_1c_ over time in large-scale observational studies should be more informative of the nature of any temporal associations than in cross-sectional studies. In this study we aimed to examine the longitudinal association of CRP and HbA_1c_ in a large prospective cohort study with repeated measurements of CRP, HbA_1c_, and other confounders, over 13 years of follow up.

## Methods

Study participants were recruited in the European Prospective Investigation into Cancer in Norfolk (EPIC-Norfolk) study. Details of this study have been previously described [8]. Briefly, 25,639 men and women, aged 40–79, resident in Norfolk, England were recruited to the study using general practice age and sex registers. The age-sex structure of the participants recruited in the study was approximately similar to the UK population age-sex structure according to registers in the United Kingdom. Study participants attended the baseline health examination between 1993 and 1997, a second health examination at around 4 years, and a third health examination around 13 years after recruitment. The EPIC-Norfolk study was approved by the Norfolk Local Research Ethics Committee, and all volunteers gave written informed consent.

At the clinic visits, extensive demographic, medical, lifestyle, family history and dietary data were collected by asking participants to complete a health and lifestyle questionnaire. Smoking status was categorized into current-, former-, and never-smokers, and alcohol consumption was computed as units per week. Medication use (including use of corticosteroids and postmenopausal hormone replacement therapy (HRT)) was assessed by self-report on the health and lifestyle questionnaire and by examining medications brought by the participants to the clinic visit. Habitual physical activity was assessed using the EPIC-validated short physical activity questionnaire by combining levels of occupational and leisure-time physical activity into 4 categories [9].

Participants were excluded from the present analysis if they were diagnosed with diabetes by a physician at baseline, or were taking diabetes medication. Prevalent and incident cases of type-2 diabetes were either ascertained by self-report of diabetes diagnosed by a physician, as well as by self-report of new administration of diabetes medication at follow up provided that it was verified by medical records, or by record linkage with external sources independent of individuals’ participation in follow up health examinations or postal questionnaires. External sources used to ascertain diabetes cases include listing with general practice diabetes registers, local hospital outpatient diabetes registers, hospital admissions information that screened for diabetes related conditions, and Office for National Statistics mortality data with coding for diabetes.

At the clinic visits, trained nurses took anthropometric measurements on individuals in light clothing without shoes. Height was measured to the nearest millimetre using a free standing stadiometer and weight was measured to the nearest 100 g using digital scales. Body mass index (BMI) was calculated as weight in kilograms divided by the square of height in metres. Waist circumference was measured to the nearest millimetre using a D-loop tape measure.

HbA_1c_ (%) was measured in fresh EDTA blood samples by using high-performance liquid chromatography at baseline and second health examination using the BioRad Diamat Automated Glycosylated Haemoglobin Analyser (Hemel Hempstead, United Kingdom), and at the third health examination using the Tosoh Automated Glycohemoglobin Analyzer.

Blood samples used for measurement of CRP at baseline were centrifuged at 2,100 g for 15 min at 4 °C and then kept frozen in −80 °C freezers until being thawed in 2008 for assaying CRP. Serum high-sensitivity CRP (mg/l) was measured using the Olympus AU640 chemistry analyzer (Olympus Diagnostics, United Kingdom). CRP was not measured at the second health examination, but was measured at the third health examination in fresh blood samples using the Siemens Dimension clinical chemistry analyzer (Siemens Dimension clinical chemistry analyzer, Newark, Delaware, US).

### Statistical analysis

Serum CRP and HbA_1c_ levels were analysed as continuous and categorical variables in separate analyses. Serum CRP had a right skewed distribution and for continuous analysis was log-transformed to obtain a normal distribution. For categorical analyses CRP was divided into 4 clinically relevant categories using cut-points 1, 3, and 10 mg/l.

In cross-sectional analysis using baseline data, multiple linear regression analysis was used to examine mean difference in HbA_1c_ across CRP categories. Analyses were adjusted for age, sex, BMI, waist circumference, smoking, physical activity, alcohol intake, corticosteroid medication, and for women only, menopausal status and HRT use. Missing values for categorical variables were coded as such and thus individuals with missing data were not excluded from the analyses.

In longitudinal analyses, linear mixed models were used to examine the rate of change of HbA_1c_ across baseline CRP categories. The rate of change of HbA_1c_ across categories of CRP was modelled as a fixed-effect interaction between follow up time (years) and CRP categories, allowing for subject-specific random effects for the intercept and time coefficients. Statistical significance of differences in longitudinal rates of change in HbA1c levels by baseline CRP levels was assessed based on *P*-value for continuous interaction between baseline log_e_-CRP and time, with subject-specific random effects for the intercept and time. The magnitude of longitudinal change in HbA_1c_ associated with a standard deviation (SD) longitudinal increase in log_e_-CRP (corresponding to a three-fold increase in original CRP (mg/l) values) over time was calculated by fitting a linear mixed model allowing for random subject specific slopes for log_e_-CRP and random subject specific intercepts. All covariates were treated as time-varying in the longitudinal analysis to account for changes in life-style and anthropometric factors over time.

Linear mixed models treat missing data as “missing at random”. However, loss to follow-up in our study was likely to be due to higher morbidity and mortality (N = 1,074 (14 %) of participants included in the analysis at baseline died during follow-up), both of which are associated with CRP and HbA_1c_ measures. Therefore, to minimize selection bias, we performed a complete case analysis in which we restricted the longitudinal analyses to participants that had adequate data for analysis at baseline and at least one follow-up health examination. The analyses were also repeated stratified by sex and BMI. Since the associations were similar in sex stratified analyses, all subsequent results are presented as sex-combined. All analyses were performed using Stata 12.0 (StataCorp. College Station, TX).

## Results

A total of 14,228 participants who were free of diabetes (as ascertained from self-report or medical records), had data on CRP at baseline, and at least one measurement of HbA_1c_, were included in the present analysis. The mean age of participants at baseline was 58 years (SD = 9) and 44 % were male (Table 1). The mean (SD) duration of follow up was 3.7 (0.7) and 13.1 (1.8) years at the second and third examinations respectively. Among participants that attended the baseline and follow-up health examinations, median (inter-quartile range) CRP (mg/l) at baseline and 13 years was 1.4 (0.7-3.0) and 2.0 (1.3-3.4) respectively. Mean (standard deviation (SD)) HbA_1c_ (%) at baseline, 4, and 13 years were 5.3 (0.7), 5.5 (0.5), and 5.8 (0.6) respectively.

**Table 1:** Baseline characteristics of European Prospective Investigation into Cancer -Norfolk cohort participants by categories of C-reactive protein and in total recruited in 1993–1997*

		All participants	C-reactive protein (mg/L)			
			≤1	1.1-3	3.1-10	>10
		*n* = 14,228	*n* = 5,460	*n* = 5,249	*n* = 2,927	*n* = 592
Age		58.0 (9.0)	55.7 (8.8)	59.0 (8.8)	60.1 (8.8)	60.7 (8.6)
Haemoglobin A1c (%)**		5.3 (0.7)	5.1 (0.6)	5.3 (0.6)	5.4 (0.7)	5.6 (0.9)
Body mass index (Kg/m^2^)		26.1 (3.7)	24.7 (3.0)	26.5 (3.4)	27.8 (4.2)	27.5 (4.8)
Waist Circumference (cm)		87.3 (12.1)	83.6 (11.2)	88.5 (11.6)	91.5 (12.2)	91.5 (13.0)
Alcohol (units/week)		7.1 (9.1)	7.2 (8.7)	7.2 (9.5)	6.7 (9.5)	6.5 (8.6)
Sex (% male)		6,211 (43.7 %)	2,412 (44.2 %)	2,361 (45.0 %)	1,177 (40.2 %)	261 (44.1 %)
Smoking	Current	1,488 (10.5 %)	445 (8.2 %)	530 (10.2 %)	413 (14.3 %)	100 (17.0 %)
	Former	5,825 (41.3 %)	2,060 (37.9 %)	2,262 (43.4 %)	1,249 (43.1 %)	254 (43.3 %)
	Never	6,809 (48.2 %)	2,926 (53.9 %)	2,415 (46.4 %)	1,235 (42.6 %)	233 (39.7 %)
Physical activity	Inactive	3,958 (27.8 %)	1,196 (21.9 %)	1,523 (29.0 %)	1,016 (34.7 %)	223 (37.7 %)
	Moderately inactive	4,104 (28.8 %)	1,582 (29.0 %)	1,525 (29.1 %)	825 (28.2 %)	172 (29.1 %)
	Moderately active	3,370 (23.7 %)	1,433 (26.3 %)	1,214 (23.1 %)	608 (20.8 %)	115 (19.4 %)
	Active	2,796 (19.7 %)	1,249 (22.9 %)	987 (18.8 %)	478 (16.3 %)	82 (13.9 %)
Prevalent disease	Cancer	733 (5.2 %)	239 (4.4 %)	287 (5.5 %)	171 (5.8 %)	36 (6.1 %)
	Myocardial Infarction	392 (2.8 %)	87 (1.6 %)	162 (3.1 %)	107 (3.7 %)	36 (6.1 %)
	Stroke	166 (1.2 %)	41 (0.8 %)	65 (1.2 %)	48 (1.6 %)	12 (2.0 %)
Corticosteroid medication use		408 (2.9 %)	103 (1.9 %)	147 (2.8 %)	121 (4.1 %)	37 (6.3 %)
Postmenopause		6,129 (76.5 %)	2,024 (66.4 %)	2,324 (80.5 %)	1,505 (86.1 %)	276 (83.4 %)
Hormone replacement therapy (% current use)		1,751 (21.9 %)	435 (14.3 %)	658 (22.8 %)	542 (31.0 %)	116 (35.1 %)

*Values are mean (standard deviation) or number (%)

**Baseline characteristics are presented for all participants who had C-reactive protein data at baseline health examination and were included in either the cross-sectional or longitudinal analyses conducted in this study. The mean (SD) of Haemoglobin A1c presented in this table is based on a total of 7,485 participants who had Haemoglobin A1c measurement at baseline. All other baseline characteristics are presented for a total of 14,228 participants who had Haemoglobin A1c measured at a minimum of one health examination

At baseline assessment, participants with higher serum CRP levels tended to be older, had higher HbA_1c_ levels, waist circumference, and BMI, were less likely to be physically active, more likely to smoke, have a pre-existing history of disease, and more likely to take corticosteroid medication or HRT (Table 1).

**Fig. 1:**
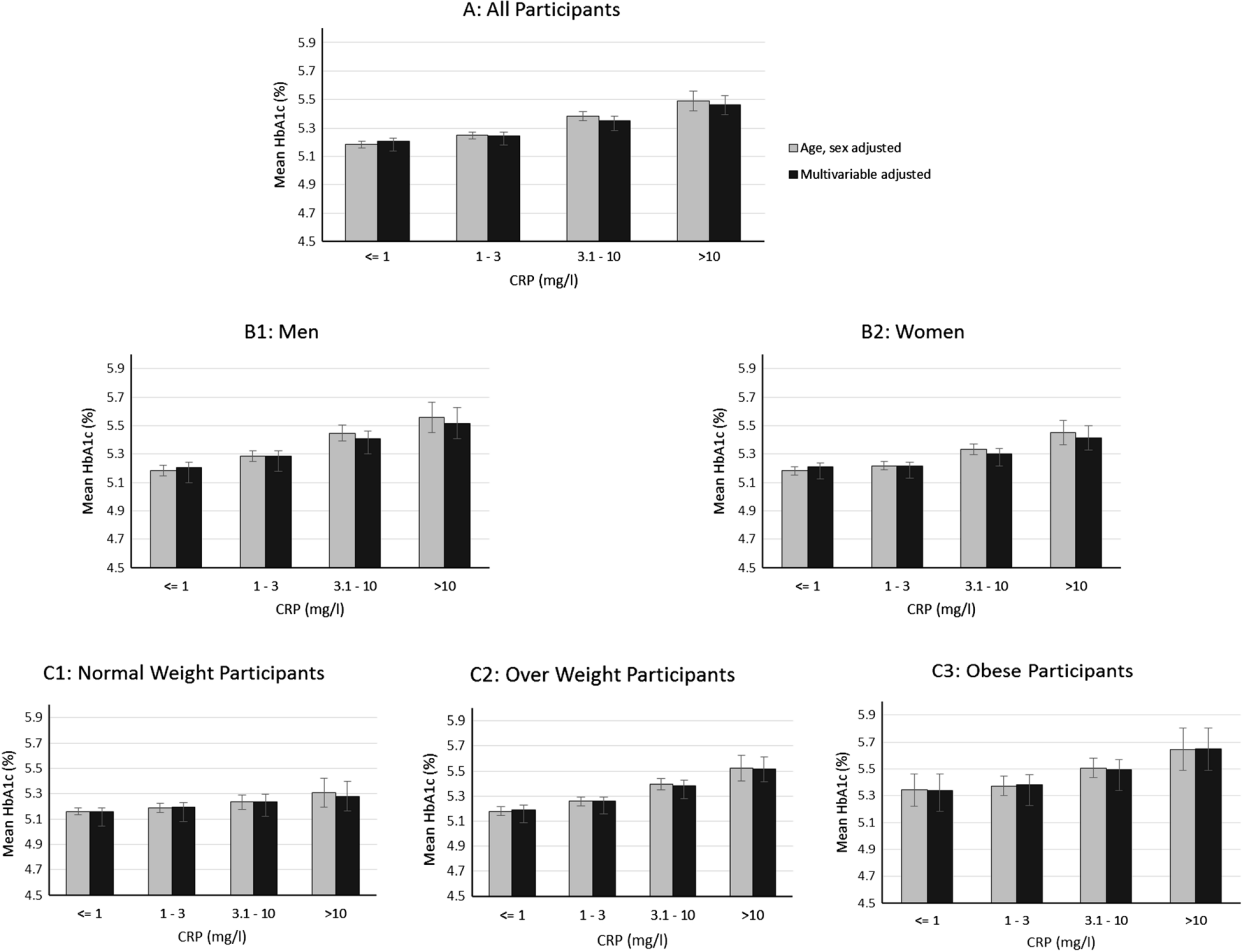
Mean haemoglobin A_1c_ (HbA_1c_) by C-reactive protein (CRP) categories measured at baseline health examination of the EPIC-Norfolk cohort study (1993–1997), among **a**) all participants and stratified by **b**) sex, and **c**) body mass index. Grey bars are mean HbA1c (%) levels adjusted for age and sex. Black bars are additionally adjusted for body mass index waist circumference, smoking, physical activity, alcohol intake, medical history of cancer, myocardial infarction, and stroke, corticosteroid medication, and in women only menopausal status and hormone replacement therapy. Normal weight refers to BMI below 25, over weight refers to BMI of 25 to 30, and obese refers to BMI over and equal to 30

In cross-sectional multiple linear regression analyses (Table 2), a 1-SD higher baseline log_e_-CRP (about three-fold higher CRP on the original scale (mg/L)) was associated with 0.09 (95 % CI 0.07, 0.10) higher HbA_1c_ (%) adjusted for age and sex. The corresponding value was 0.06 (95 % CI 0.04, 0.08) after adjustment for other covariates. Multivariable adjusted mean HbA_1c_ levels were higher in participants with higher baseline CRP levels (Fig. 1). No significant interaction by sex or BMI was observed and the positive associations persisted in stratified analyses (Fig. 1).

**Table 2:** Cross-sectional and longitudinal association of Haemoglobin A_1c_ with baseline levels of C-reactive protein in the EPIC-Norfolk cohort study (1993–2011)

	Cross-sectional association^a^		Longitudinal association^b^	
	*n* = 7,485		*n* = 4,595	
CRP levels (mg/L)	Age-sex adjusted	Multivariable adjusted	Age-sex adjusted	Multivariable adjusted
	Mean difference (95 % confidence interval)	Mean difference (95 % confidence interval)	Annual change (95 % confidence interval)	Annual change (95 % confidence interval)
**≤**1	Reference	Reference	0.043 (0.041, 0.045)	0.039 (0.031, 0.046)
1.1 – 3	0.065 (0.032, 0.098)	0.040 (0.007, 0.074)	0.045 (0.043, 0.047)	0.040 (0.032, 0.047)
3.1 – 10	0.198 (0.159, 0.238)	0.143 (0.101, 0.185)	0.048 (0.044, 0.051)	0.040 (0.031, 0.047)
>10	0.308 (0.236, 0.379)	0.256 (0.183, 0.328)	0.052 (0.045, 0.059)	0.041 (0.028, 0.054)
1-SD increase in CRP	0.086 (0.071, 0.10)	0.059 (0.046, 0.072)	0.003 (0.0002, 0.005)^c^	0.002 (−0.0007, 0.004)^c^
*P*-value	<0.001	<0.001	0.02	0.15

*CRP* C-reactive protein, *EPIC-Norfolk* European Prospective Investigation into Cancer in Norfolk, *SD* standard deviation

^a^Mean difference in HbA_1c_ (%) in each category of CRP and the reference category (CRP ≤ 1 mg/l). Multivariable cross-sectional analyses are adjusted for baseline covariates: age, sex, body mass index, waist circumference, smoking, physical activity, alcohol intake, medical history of cancer, myocardial infarction, and stroke, corticosteroid medication, and in women only menopausal status and hormone replacement therapy

^b^Annual change calculated as an interaction of baseline CRP categories with time in participants who had at least one follow-up health examination. Adjusted for baseline age and sex. Multivariable analyses are additionally adjusted for body mass index, waist circumference, smoking, physical activity, alcohol intake, medical history of cancer, myocardial infarction, and stroke, corticosteroid medication, and in women only menopausal status and hormone replacement therapy, all treated as time varying variables. Analysis is restricted to individuals who had at least two measurements of HbA_1c_

^c^Values are change in HbA_1c_ over 13 years

HbA_1c_ increased by about 0.05 (SD = 0.05) annually. In longitudinal analyses, the annualised rates of change of HbA_1c_ was significantly associated with baseline CRP levels adjusted for age, sex and follow-up time (P = 0.02 for continuous baseline log_e_ CRP*time interaction, Table 2). Annualized rates of change of HbA_1c_ were no longer significantly associated with baseline CRP levels in multivariable analysis. The results were not changed when analysis was restricted to individuals who had data for CRP, HbA_1c,_ and other covariates at baseline and third health examination (data not shown).

**Fig. 2:**
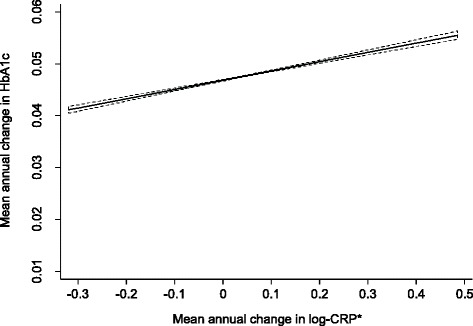
Association between the mean annual change in haemoglobin A_1c_ (HbA_1c_) and the mean annual change in C-reactive protein (CRP) measured at baseline and third health examinations in the EPIC-Norfolk cohort study (1993–2011), adjusted for baseline age and sex. *Change in log-CRP is equivalent to percentage change in CRP on the original mg/l scale. For example 0.1 units change in log-CRP is roughly equivalent to 10 % change in CRP (mg/l) values

In addition to baseline levels of CRP, change in CRP over time was also significantly and positively associated with longitudinal change in HbA_1c_ (Fig. 2). A 1-SD increment in log_e_ CRP (corresponding to 3-fold increase in CRP on the original mg/l scale) over 13 years of follow-up was associated with an increase of 0.06 (95 % CI 0.04, 0.07) in HbA_1c_ adjusted for age, sex, and follow up time. After adjustment for changes in other covariates in multivariable models, the associations attenuated but remained statistically significant, such that a three-fold increase in CRP over 13 years was associated with 0.04 (95 % CI 0.02, 0.05) increase in HbA_1c_ over the same period.

## Discussion

In this study we found a cross-sectional positive association between serum CRP and HbA_1c_. A 1-SD higher log_e_-CRP was cross-sectionally associated with approximately 0.06 higher HbA_1c_ (%). Changes in levels of serum CRP over time were also positively associated with change in HbA_1c_ levels, but to a lesser extent to that found in cross-sectional analysis; such that 1-SD increase in log_e_-CRP levels over time was associated with ~ 0.04 increase in HbA_1c_ (%) over the same period. These associations were largely independent of age, sex, BMI, waist circumference, smoking status, physical activity, alcohol intake, medical history of diseases, corticosteroid medication, and HRT use.

The cross-sectional positive association between CRP and HbA_1c_ levels has been previously shown in studies involving participants with diabetes [5] and without diabetes [4, 6, 7] among different populations and age groups. The multivariable adjusted cross-sectional associations of HbA_1c_ and log-CRP observed in the present analysis were similar to those in previous studies [4, 6, 7]. CRP has also been shown to be positively associated with incident type 2 diabetes in previous studies [1, 2]. However, to the best of our knowledge, the longitudinal association between CRP and HbA_1c_ has not been previously reported.

### Strengths and limitations

An important consideration in investigating the role of inflammation in development of diabetes and abnormal glucose metabolism is confounding by obesity. Obesity and high body fat are associated with diabetes and abnormal glucose metabolism, as well as with elevated circulating levels of CRP and other pro-inflammatory cytokines [10-12]. A strength of the present study is the large sample size and the availability of repeated measurements of all variables including BMI and waist circumference. Availability of repeated measurements reduces the possibility of measurement error and also enables us to better account for concurrent changes in confounding factors (such as BMI and waist circumference) over time and thus enabling better understanding of the association between inflammation and glucose metabolism independent of obesity.

We excluded participants who were diagnosed with diabetes by a physician or were taking diabetes medication at baseline because treatment for diabetes might affect the association between CRP and HbA_1c_. We also restricted the analyses to individuals who attended both the baseline and follow up health examinations, and had adequate data required for analysis, to avoid potential bias due to lower survival in those with higher CRP and HbA_1c_ levels.

A limitation of the present study was that CRP at baseline was measured in serum samples that were kept frozen for about 15 years [13]. However, previous studies have shown that CRP is relatively stable in frozen plasma [14]. CRP at the third health examination was measured in fresh serum samples using a different chemistry analyzer. Difference in the methods and assays used for measurement of CRP and HbA_1c_ across different time points may have increased the measurement error. However, the intra-class correlation coefficient between measurements of HbA_1c_ (0.62) was considerably higher than that of systolic blood pressure (0.39) and LDL-cholesterol (0.28), and the intra-class correlation coefficient between the two measurements of log CRP (0.39) was similar to that of systolic blood pressure, and higher than that of LDL cholesterol measured at the same time points in the EPIC-Norfolk study (unpublished data). Similar results were reported in other studies [10, 15].

### Biological mechanisms

Although we observed a temporal positive association between change in CRP and change in HbA_1c_, it is not possible from an observational study to infer causality. Knowledge of biological mechanisms justifies the plausibility of association. Tumour necrosis factor alpha (TNF-α), and interleukin-6 (IL-6) are pro-inflammatory cytokines over expressed in adipose tissue. IL-6 and TNF-α in turn stimulate hepatic production of CRP [16]. The link between obesity inflammation, and abnormal glucose metabolism may in part be related to increased production of pro-inflammatory cytokines such as IL-6 and TNF-α in adipose tissue. TNF-α is suggested to induce insulin resistance [17]. Interleukin-6 and CRP are also shown in animal studies to impair insulin signalling and insulin sensitivity mainly through insulin receptor substrate phosphorylation [18-22]. Further investigation of the association between CRP and markers of insulin resistance such as HOMA-IR is required to underpin this hypothesis.

Higher levels of CRP and leptin were shown to be associated with increased metabolic syndrome components [23]. On the other hand, plasma leptin levels are associated with vascular endothelial function in overweight patients with type 2 diabetes [24] and leptin was found to be directly related to CRP independently of BMI and other confounding factors especially in men [25]. These findings are consistent with and support a role of CRP and inflammation in abnormal glucose metabolism.

CRP is a marker of an age-related pro-inflammatory process described as “inflamm-aging” [26-28]. According to this theory, long-term exposure to a variety of antigens (infection, food, etc.) results in progressive filling of the immune system by activated immune cells and pro-inflammatory cytokines. This process may lead to a reduced capacity to respond to infectious and stress factors later in life. It may additionally lead to a pro-inflammatory state resulting from increased activated immune cells and pro-inflammatory cytokines. The pro-inflammatory state is suggested to cause long-term tissue damage and an increased susceptibility to non-infectious diseases, such as diabetes and impairments in glucose metabolism, where immunity and inflammation play a role [26-29]. The studies exploring the association between CRP related genes and HbA_1c_ levels or incident diabetes do not support a causal role for CRP [30]. Nevertheless, CRP genotype polymorphisms are associated with a small difference (about 60 %) in serum CRP levels, and of insufficient magnitude for an association with incident diabetes to be detected. Moreover, the negative findings of the studies regarding CRP related genes and diabetes risk do not preclude a causal role for the inflammatory process (of which CRP is an indicator) in development of diabetes.

It remains unclear whether systemic inflammation has a causal role in development of diabetes and related conditions, or whether it is a case of reverse causation, i.e., disturbance in glucose metabolism being accountable for causing systemic inflammation. Previous studies have shown that hyperglycaemia induces secretion of pro-inflammatory cytokines and acute phase reactants by adipocytes and monocytes [31,32]. Moreover, the possibility of the temporal association between CRP and HbA_1c_ being due to residual confounding by unmeasured genetic, lifestyle, or environmental factors cannot be ruled out.

## Conclusions

The associations observed in the present study provide observational evidence suggesting that systemic inflammation may potentially have an etiologic role in development of diabetes and may be involved in the link between elevated HbA_1c_ levels and development of diabetic complications such as cardiovascular diseases. Further biological and interventional studies are required to elucidate these hypotheses.
